# Combined effect of cognitive dysfunction and sleep disturbance on mortality risk: NHANES 2011–2014

**DOI:** 10.1186/s12991-025-00593-7

**Published:** 2025-09-02

**Authors:** Tian-Shin Yeh, Yi-Chen Huang, Shu-Chuan Ho, Co-Yih Siow, Hsin-Chien Lee, Jose I Recio-Rodriguez, Jia-Ying Sung, Jowy Tani

**Affiliations:** 1 Department of Physical Medicine and Rehabilitation, School of Medicine, College of Medicine, Taipei Medical University, Taipei, Taiwan; 2 Department of Physical Medicine and Rehabilitation, Shuang-Ho Hospital, Taipei Medical University, New Taipei City, Taiwan; 3 Department of Neurology, Wan Fang Hospital, Taipei Medical University, No.111, Sec. 3, Xinglong Rd., Wenshan Dist, Taipei City, 116 Taiwan; 4 Taipei Neuroscience Institute, Taipei Medical University, Taipei, Taiwan; 5 School of Respiratory Therapy, College of Medicine, Taipei Medical University, Taipei, Taiwan; 6 Division of Pulmonary Medicine, Department of Internal Medicine, Shuang Ho Hospital, Taipei Medical University, New Taipei City, Taiwan; 7 Department of Physical Medicine and Rehabilitation, Taipei Medical University Hospital, Taipei Medical University, Taipei, Taiwan; 8 Department of Psychiatry, School of Medicine, College of Medicine, Taipei Medical University, Taipei, Taiwan; 9 Department of Psychiatry, Taipei Medical University Hospital, Taipei Medical University, Taipei, Taiwan; 10 Departamento de Enfermería y Fisioterapia, Universidad de Salamanca, Salamanca, 37007 España; 11 Instituto de Investigación Biomédica de Salamanca (IBSAL), Salamanca, 37007 España; 12 Unidad de Investigación en Atención Primaria de Salamanca (APISAL), Salamanca, 37005 España; 13 Red de Investigación en Cronicidad, Atención Primaria y Promoción de la Salud (RICAPPS), Madrid, España; 14 Department of Neurology, School of Medicine, College of Medicine, Taipei Medical University, Taipei, Taiwan; 15 Ph.D. Program in Medical Neuroscience, Taipei Medical University, Taipei, Taiwan; 16 College of Biomedical Engineering, Taipei Medical University, Taipei, Taiwan

**Keywords:** Cognitive dysfunction, Mortality, National health and nutrition examination survey (NHANES), Sleep disturbance

## Abstract

**Background:**

Both cognitive dysfunction and sleep disturbances are individually linked to heightened risks of chronic illnesses and mortality. However, their combined impact on all-cause and cardiovascular mortality remains underexplored.

**Methods:**

This study utilized data from the National Health and Nutrition Examination Survey (NHANES) from 2011 to 2014, focusing on participants aged ≥ 60 years who completed cognitive tests and sleep-related questionnaires. Cognitive function was evaluated using three standardized tests: The Consortium to Establish a Registry for Alzheimer’s Disease (CERAD), the Animal Fluency Test, and the Digit Symbol Substitution Test. Participants with global cognitive z-scores below − 1 were classified as having low cognitive function. Sleep disturbance was identified based on self-reported diagnoses of sleep disorders or complaints of trouble sleeping. Mortality data were sourced from the National Death Index. Cox proportional hazards regression was used to calculate adjusted hazard ratios (aHR) for all-cause and cardiovascular mortality, with adjustments for potential confounders.

**Results:**

A total of 3,170 participants ≥ 60 years of age were included for analysis. Participants with low cognitive function alone had an adjusted hazard ratio (aHR) of 1.59 (95% CI: 1.12–2.26) for all-cause mortality. The risk increased to an aHR of 1.73 (95% CI: 1.07–2.79) when both low cognitive function and sleep disturbances were present. Stratified analyses revealed that the associations between cognitive function, sleep disturbance, and mortality risks varied across sex, BMI, and chronic kidney disease status.

**Conclusions:**

The combination of low cognitive function and sleep disturbances is associated with a higher risk of all-cause and cardiovascular mortality, exceeding the risk of either condition alone. These findings emphasize the need to consider both factors together when assessing mortality risk in older adults.

**Supplementary Information:**

The online version contains supplementary material available at 10.1186/s12991-025-00593-7.

## Introduction

Cognitive dysfunction refers to impairments in memory, attention, and problem-solving abilities, which can significantly impact daily life. It may arise from various neurological conditions, including Alzheimer’s disease, vascular brain injury, and other neurodegenerative disorders. In many cases, cognitive dysfunction progresses gradually, often progressing to mild cognitive impairment (MCI) or dementia, both of which can severely impact daily functioning [1]. MCI alone affects about 19.0% of adults aged ≥ 50 years [2]. The World Health Organization reports that over 55 million people globally suffer from dementia, with nearly 10 million new cases diagnosed each year [3]. These cognitive disorders significantly affect memory, attention, and problem-solving abilities, contributing to a diminished quality of life and increased mortality risk. Moreover, research has consistently linked cognitive dysfunction to heightened risks of cardiovascular disease and premature death [4, 5].

Sleep disturbances encompass various conditions such as insomnia, sleep-disordered breathing, and irregular sleep duration or timing, all of which can adversely affect brain health and overall functioning [6]. Studies suggest that approximately 30% of adults experience some form of sleep disruption, with conditions like insomnia affecting around 10% of the population [7, 8]. Sleep disturbances are associated with a range of risks, including increased vulnerability to mental health disorders and physical health issues [9, 10].

Emerging research suggests a bidirectional relationship between cognitive dysfunction and sleep disturbances. Poor sleep quality has been associated with accelerated progression of cognitive decline, impair cognitive processes like memory consolidation [11] and synaptic plasticity by preventing the clearance of neurotoxic substances like beta-amyloid [12]. Conversely, cognitive impairment can damage brain regions controlling the sleep-wake cycle, resulting in disrupted sleep patterns [13]. Furthermore, the coexistence of both conditions may have compounded effects on health, driven by shared mechanisms such as chronic inflammation, hormonal dysregulation, and impaired neurovascular function [14, 15].

Although the individual clinical impacts of these conditions, such as mortality, are well recognized, their combined effect on mortality, especially in the context of cardiovascular disease, has not been thoroughly investigated. This study examined the joint effects of cognitive dysfunction and sleep disturbances on all-cause and cardiovascular-related mortality using data from the National Health and Nutrition Examination Survey (NHANES). By leveraging this nationally representative dataset, the research aims to provide new insights into the compounded risks posed by these conditions and inform potential interventions and health policies aimed at reducing mortality in vulnerable populations.

## Methods

### Data source

The present study analyzed data from The National Health and Nutrition Examination Survey (NHANES) database, a nationally representative survey conducted by the Centers for Disease Control and Prevention (CDC), National Center for Health Statistics (NCHS) in the USA. (http://www.cdc.gov/nchs/nhanes/). The survey aimed to assess the health and nutritional status of adults and children across the USA. It employs a complex, multistage approach to gather and analyze data representative of the national, non-institutionalized US population. The National Center for Health Statistics (NCHS) releases the data for research and grants usage permissions to researchers. Participants in NHANES undergo a household interview and are subsequently invited to a mobile examination center (MEC) for a comprehensive examination, which includes a physical assessment, specialized measurements, and laboratory tests. Consequently, an evaluation of subjects within the NHANES database is reliable and multidimensional and can be equated to a population-level assessment.

### Ethics statements

The NCHS Research Ethics Review Board (ERB) reviewed and approved NHANES, and all survey participants provided written informed consent. Therefore, no further ethical approval and informed consent was required to perform this secondary analyses. For additional information see the NHANES website for NCHS Research ERB Approval (https://www.cdc.gov/nchs/nhanes/about/erb.html). All NHANES data released by the NCHS are de-identified and the data remain anonymous during data analysis.

### Study population

The present study utilized data from the NHANES database from 2011 to 2014, focusing on participants aged ≥ 60 years. This specific age group and study cycle were chosen because cognitive function tests were administered exclusively to participants within this demographic population during these years. The inclusion criteria for the cohort required participants to have documented education levels, completed sleep-related questionnaires, and performance on at least one of the three cognitive tests conducted by NHANES, as detailed in the subsequent section. Participants without information of sex and sample weight were excluded from the primary cohort.

Participants were divided into four groups based on the presence of low cognitive function and sleep disturbances: C(-)S(-) for those without low cognitive function and without sleep disturbances; C(-)S(+) for those without low cognitive function but with sleep disturbances; C(+)S(-) for those with low cognitive function and without sleep disturbances; C(+)S(+) for those with both low cognitive dysfunction and sleep disturbances.

This study followed the Strengthening the Reporting of Observational Studies in Epidemiology (STROBE) guideline.

### Ascertainment of low cognitive function

To assess cognitive function, NHANES uses three cognitive tests: the Consortium to Establish a Registry for Alzheimer’s Disease (CERAD) test, the Animal Fluency Test, and the Digit Symbol Score Test (DSST). For CERAD, the test is the sum of three learning trials and one recall trial. The Animal Fluency Test and DSST are additional measures of cognitive performance. Since education level is a significant factor influencing cognitive performance, participants’ scores were adjusted for education level. Individuals were grouped into five education categories based on final level reached: <9th grade, 9 to 11th grade, high school graduate or GED, some college or associate’s degree, and college graduate or above. Within each education group, participants’ test scores were standardized to have a mean of 0 and a standard deviation of 1, yielding education-dependent z-scores. A global cognitive score was calculated as the average of these standardized scores from all three tests [16]. Individuals with global or individual cognitive scores less than − 1 were classified as having low cognitive function.

### Ascertainment of sleep disturbance

Participants were categorized as having sleep disturbances based on their responses to the following questions: “Have you or has the survey participant ever reported to a doctor or other health professional that you have or they have trouble sleeping?” or “Have you or has the study participant ever been told by a doctor or other health professional that you have or they have a sleep disorder?” A “Yes” response to either question led to classification under “sleep disturbance [15].

### Mortality

Mortality information for NHANES participants is sourced from the National Center for Health Statistics (NCHS) via the Public-Use Linked Mortality File, which links to the National Death Index (NDI)—a comprehensive database of all U.S. deaths—up to December 31, 2019. The cause of death for participants is determined according to the International Classification of Diseases and Related Health Problems, Tenth Revision (ICD-10). Deaths attributed to cardiovascular diseases are specifically classified under ICD-10 codes I00–I09, I11, I13, and I20–I51, defining cardiovascular disease (CVD) mortality.

### Demography and covariates

Data of participants’ age, sex, race/ethnicity, education level, poverty income ratio, were obtained by standard questionnaires through in-person home interviews conducted by trained interviewers using the Family and Sample Person Demographics questionnaires and the Computer-Assisted Personal Interviewing (CAPI) system (Confirmit Corp. New York, USA). Collected data were weighted according to the NHANES protocol. All biological samples were collected by trained phlebotomists and technicians, transported under strict cold chain protocols, and maintained by the CDC’s Division of Laboratory Sciences for secure long-term storage.

Body mass index (BMI) values were derived from examination measurements in the NHANES database, calculated as body weight in kilograms divided by the square of height in meters. Body weight was measured using an electronic load cell scale, while standing height was gauged with a fixed stadiometer. BMI categories were defined as follows: underweight (BMI < 18.5), normal (BMI 18.5 to < 25), overweight (BMI 25 to < 30), and obese (BMI ≥ 30).

Diabetes mellitus (DM) was defined by the answer of questionnaire and results of laboratory analysis. The questionnaire considered were “Self-reported of having a diagnosis of diabetes by a doctor” and “Now using anti-diabetic drugs”, Laboratory results were glycosylated hemoglobin A1c (HbA1c) level of ≥ 6.5%, fasting glucose (FPG) ≥ 126 mg/dL, and oral glucose tolerance test (OGTT) ≥ 200 mg/dL. Insulin use was confirmed through participant responses to a questionnaire.

The determination of hypertension was based on responses to two specific questionnaire items: “Have you been told on two or more different visits that you have hypertension?” and “Are you currently taking medication to reduce your blood pressure?” Additionally, an indication of hypertension was confirmed through measured systolic blood pressure of 140 mmHg or greater and diastolic blood pressure of 90 mmHg or greater.

The smoking status of participants was categorized based on their lifetime smoking history and current smoking habits as follows: individuals who have smoked < 100 cigarettes in their lifetime were classified as non-smokers; those who have smoked > 100 cigarettes but do not currently smoke were classified as former smokers; and those who have smoked > 100 cigarettes and responded “yes” to the question, “Do you smoke now?” were classified as current smokers.

Chronic Obstructive Pulmonary Disease (COPD) was determined by a positive answer of any of the question: “Ever told you had COPD?”; “Ever told you had emphysema?”; and “Ever told you had chronic bronchitis?”.

Chronic kidney disease (CKD) was defined by estimated glomerular filtration rate (eGFR) < 60 mL/min/1.73 m². eGFR was calculated by the following equation: *175 × (serum creatinine)-1.154 × (Age)-0.203 × (0.742 if female) × (1.212 if African American).*

CVD encompassed coronary heart disease, angina pectoris, heart attack, stroke, and congestive heart failure.

A history of cancer was ascertained through responses to the questionnaire.

The levels of total cholesterol, triglycerides, high-density lipoprotein cholesterol (HDL-c), albumin, HbA1c, and the counts of platelets, lymphocytes, and neutrophils were obtained from the NHANES laboratory data.

### Statistical analysis

NHANES employs a complex, multistage, probability sampling design to ensure national representation. Analyses incorporated sampling weights, pseudo-stratum, and pseudo-cluster as provided by NHANES, following guidance from the NCHS. For continuous variables, weighted means and standard errors were presented; for categorical variables, unweighted counts and weighted proportions were reported. Differences in means between groups were compared by using the SURVEYREG procedure for continuous variables, while differences in proportions were assessed with the Rao-Scott chi-square test. Cox proportional hazards regression was used to calculate hazard ratios (HR) and 95% confidence intervals (CI) for estimating associations between low cognitive function, sleep disturbance, all-cause mortality, and CVD mortality. Multivariable regression was adjusted for related variables significant in univariate analysis (*p* < 0.05), except for the laboratory values, variables with excessive missing data (more than 20%), and variables that were highly correlated with included factors (different models for different outcome). Sensitivity analyses were conducted by including all variables in the multivariable regression model to assess the robustness of the results (Table 1). We constructed Kaplan–Meier survival curves and used the log-rank test to compare overall and CVD-specific survival between groups categorized by cognitive function and sleep status. A two-sided p-value of < 0.05 was considered statistically significant. All analyses accounted for the NHANES’s complex survey design to ensure accurate national estimates. Statistical analyses were conducted using SAS statistical software (version 9.4, SAS Inc., Cary, NC, USA).

**Table 1:** Characteristics of the study population

Characteristics	Total	Cognitive function and sleep status				
	n = 3,170	C(-), S(-)	C(-), S(+)	C(+), S(-)	C(+), S(+)	
		n = 1,436	n = 712	n = 708	n = 314	*p*-value
**All-cause mortality**	685 (19.3)	219 (13.1)	114 (14.1)	228 (36.7)	124 (41.4)	**< 0.001**
**CVD mortality**	229 (6.1)	74 (3.9)	31 (4.1)	79 (12.8)	45 (14.6)	**< 0.001**
**Demography**						
**Age, years**	69.4 ± 0.2	68.3 ± 0.2	68.0 ± 0.3	73.3 ± 0.3	73.4 ± 0.4	**< 0.001**
60–69	1670 (54.7)	858 (61.4)	433 (64.8)	256 (28.4)	123 (29.7)	**< 0.001**
70–79	946 (29.6)	398 (28.1)	194 (24.5)	248 (38.7)	106 (38.4)	
80+	554 (15.7)	180 (10.5)	85 (10.7)	204 (32.8)	85 (31.9)	
**Sex**						**< 0.001**
Male	1537 (45.2)	705 (48.6)	296 (38.2)	406 (50.6)	130 (37.6)	
Female	1633 (54.8)	731 (51.4)	416 (61.8)	302 (49.4)	184 (62.4)	
**Race/ethnicity**						**< 0.001**
Non-Hispanic White	1466 (78.3)	692 (81.2)	396 (83.8)	240 (63.5)	138 (69.8)	
Non-Hispanic Black	773 (8.9)	300 (6.8)	139 (6.2)	240 (17.0)	94 (15.9)	
Hispanic	324 (3.9)	143 (3.4)	61 (2.5)	86 (7.1)	34 (5.1)	
Others	607 (8.9)	301 (8.6)	116 (7.5)	142 (12.4)	48 (9.2)	
**Education**						0.143
High school and above	2299 (82.8)	1045 (83.7)	532 (83.7)	482 (78.6)	240 (82.9)	
Never attend high school	871 (17.2)	391 (16.3)	180 (16.3)	226 (21.4)	74 (17.1)	
**BMI, kg/m** ^**2**^						**0.012**
Normal	791 (24.7)	382 (25.4)	153 (21.9)	188 (27.0)	68 (25.0)	
Underweight	47 (1.5)	19 (1.5)	7 (0.6)	17 (2.7)	4 (1.8)	
Overweight	1091 (36.1)	501 (37.7)	228 (32.3)	257 (37.4)	105 (36.4)	
Obese	1180 (37.8)	516 (35.3)	318 (45.1)	221 (33.0)	125 (36.9)	
Missing	61	18	6	25	12	
**Poverty income ratio**						**< 0.001**
Not poor	2364 (90.0)	1126 (92.5)	548 (91.7)	466 (80.7)	224 (86.0)	
Poor	533 (10.0)	192 (7.5)	111 (8.3)	164 (19.3)	66 (14.0)	
Missing	273	118	53	78	24	
**Cigarette smoking**						0.138
Never	1574 (49.7)	726 (51.7)	328 (44.8)	368 (53.5)	152 (46.7)	
Former	1190 (39.2)	521 (37.0)	299 (44.0)	251 (36.8)	119 (41.8)	
Current smoker	403 (11.1)	188 (11.3)	83 (11.3)	89 (9.7)	43 (11.6)	
Missing	3	1	2	0	0	
Physical exercise	1496.8 ± 64.1	1595.7 ± 84.9	1395.0 ± 96.9	1583.6 ± 131.8	863.5 ± 77.7	< 0.001
< 500	507 (28.6)	225 (27.0)	124 (27.4)	100 (29.7)	58 (48.5)	0.003
>= 500	1131 (71.4)	575 (73.0)	257 (72.6)	234 (70.3)	65 (51.5)	
Missing	1532	636	331	374	191	
**DM**	1062 (27.2)	409 (23.1)	253 (30.8)	258 (29.2)	142 (37.2)	**< 0.001**
HbA1c, % (Missing = 121)	6.0 ± 0.02	5.9 ± 0.04	6.0 ± 0.05	6.0 ± 0.1	6.1 ± 0.1	**0.038**
≥ 8	178 (4.7)	64 (3.6)	40 (5.5)	50 (5.7)	24 (7.5)	0.127
< 8	2871 (95.3)	1324 (96.4)	653 (94.5)	618 (94.3)	276 (92.5)	
Now using anti-diabetic drugs-Insulin	216 (5.2)	56 (3.3)	52 (6.0)	55 (5.9)	53 (13.8)	**< 0.001**
**Hypertension**	2249 (67.0)	958 (63.0)	507 (66.8)	541 (74.6)	243 (78.8)	**< 0.001**
**CVD**	713 (22.0)	229 (16.1)	183 (24.9)	187 (28.0)	114 (37.7)	**< 0.001**
**Cancer history**	638 (24.1)	285 (23.6)	160 (25.9)	114 (19.6)	79 (29.6)	0.07
**CKD** (Missing = 187)	728 (24.2)	263 (19.1)	163 (23.9)	179 (31.1)	123 (45.3)	**< 0.001**
eGFR, mL/min/1.73 m² (Missing = 187)	72.6 ± 0.4	74.2 ± 0.6	73.0 ± 0.8	69.4 ± 0.9	66.7 ± 1.8	**< 0.001**
**Statins**	1352 (43.8)	559 (40.0)	342 (48.7)	291 (41.5)	160 (55.2)	**< 0.001**
**Diuretics**	2614 (83.7)	1242 (87.3)	574 (82.3)	568 (80.3)	230 (72.8)	**< 0.001**
**Laboratory measures**						
Total cholesterol, mg/dL (Missing = 188)	191.9 ± 1.2	195.3 ± 1.9	191.8 ± 2.2	187.0 ± 2.2	179.6 ± 3.4	**< 0.001**
Triglyceride, mg/dL (Missing = 1674)	125.4 ± 3.6	123.1 ± 3.8	135.2 ± 5.4	114.9 ± 4.2	125.8 ± 8.3	**0.039**
HDL-c, mg/dL (Missing = 176)	55.5 ± 0.7	56.3 ± 0.8	54.7 ± 0.9	54.7 ± 0.9	54.4 ± 1.6	0.261
Platelet, 10^9^/L (Missing = 113)	224.2 ± 1.6	225.5 ± 1.4	230.4 ± 4.4	212.4 ± 3.3	217.9 ± 5.6	**0.003**
Lymphocyte, 10^9^/L (Missing = 119)	1.9 ± 0.03	1.9 ± 0.03	2.0 ± 0.1	1.9 ± 0.1	1.9 ± 0.1	0.167
Neutrophil, 10^9^/L (Missing = 119)	4.2 ± 0.04	4.1 ± 0.1	4.3 ± 0.1	4.3 ± 0.1	4.5 ± 0.1	**0.023**
Albumin, g/dL (Missing = 187)	42.1 ± 0.1	42.3 ± 0.1	42.1 ± 0.1	41.4 ± 0.2	41.3 ± 0.2	**0.002**

Abbreviations: BMI, body mass index; CVD, cardiovascular disease; CKD, chronic kidney disease; eGFR, estimated glomerular filtration rate; HbA1c, glycated hemoglobin; HDL-c, high-density lipoprotein-cholesterol

Categorical variables are presented as unweighted counts (weighted percentage), and continuous variables as mean ± SE. Group comparisons were performed using Rao–Scott chi-square test or survey-weighted linear regression, as appropriate

P-values < 0.05 are shown in bold

## Results

### Study population selection

**Fig. 1:**
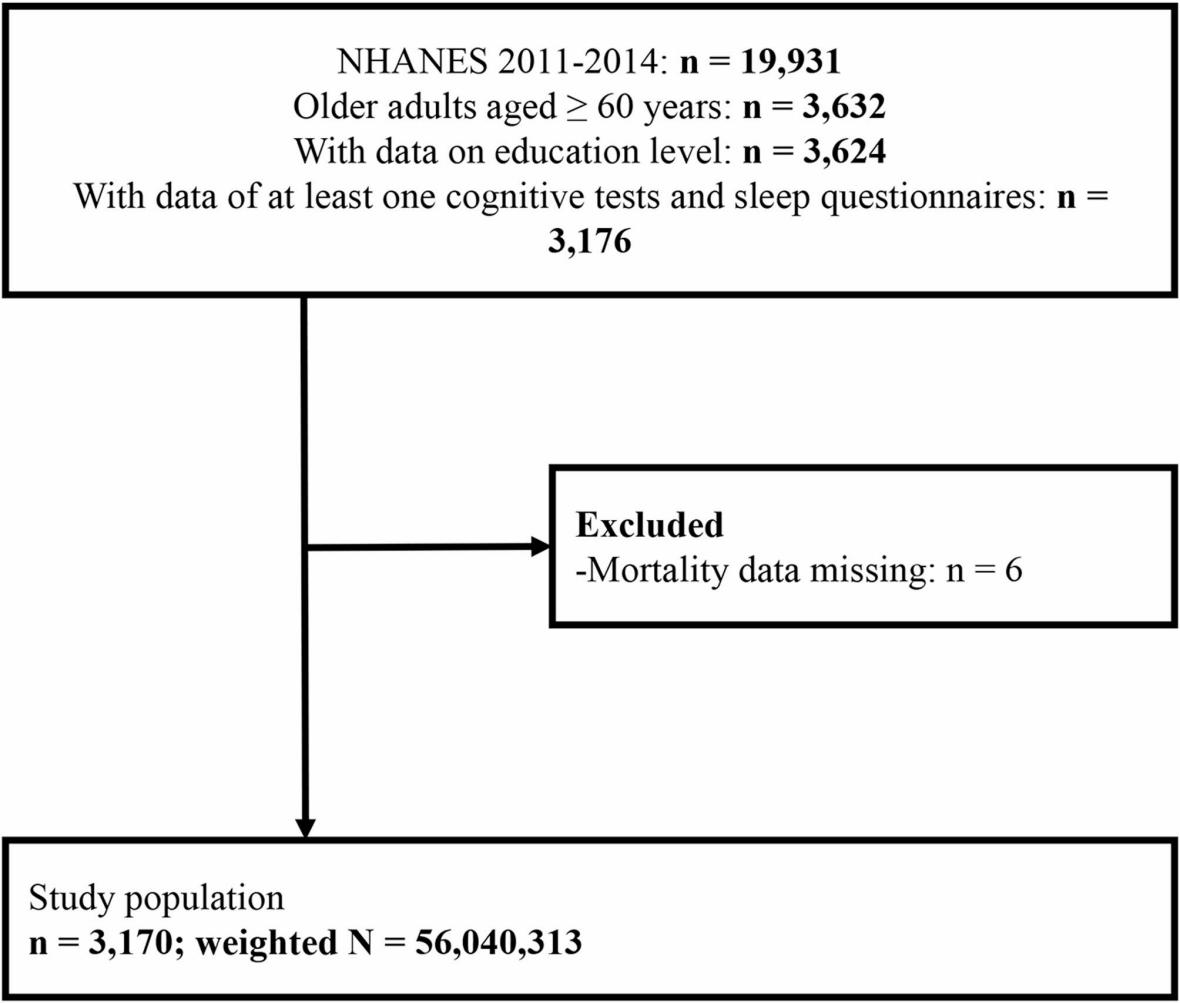
Flow diagram of study population selection process

A total of 19,931 subjects from the 2011 to 2014 NHANES cycles were analyzed (Fig. 1). Of these, 3,176 participants aged ≥ 60 years had available data on education level, at least one of the three cognitive tests, and responses to sleep-related questionnaires. Participants with missing information on mortality (*N* = 6) were excluded. Finally, 3,170 participants were selected in the study. Based on the sample weights provided by NHANES, this sample represents a population of 56,040,313 participants in the entire US (Fig. 1).

### Characteristics of the study population

The mean age of the study population was 69.4 years, the majority were female (54.8%), and were non-Hispanic White (78.3%) (Table 1). Significant differences were identified in age, sex, race/ethnicity, BMI, poverty income ratio, physical activity, depression, DM, hypertension, CVD, CKD, statins, diuretics, total cholesterol, triglycerides, platelets, neutrophils, and albumin across the four study groups. Patients with both low cognitive function and sleep disturbance exhibited the highest proportions of all-cause mortality (41.4% vs. 13.1–36.7%, *p* < 0.001) and CVD mortality (14.6% vs. 3.9–12.8%) (Table 1).

### Joint associations between low cognitive dysfunction, sleep disturbance, and mortality outcomes

For the multivariable analysis, univariate analyses were performed to identify potential confounders associated with cognitive dysfunction, sleep disturbance, and mortality outcomes. The univariate results are presented in Supplemental Tables 1 and Supplemental Table 2.

For all-cause mortality, the potential confounders included age (continuous), race, education, BMI, poverty income ratio, cigarette smoking, diabetes mellitus (DM), hypertension, history of cardiovascular disease (CVD), cancer history, and chronic kidney disease (CKD). For CVD-related mortality, the identified confounders were age (continuous), race, education, poverty income ratio, DM, hypertension, history of CVD, and CKD. These variables were included in the multivariable analysis.

After adjustment in multivariable analysis, participants with low cognitive function but without sleep disturbance (adjusted hazard ratio [aHR] = 1.59, 95% confidence interval [10]: 1.12–2.26, *p* = 0.012), and those with both low cognitive function and sleep disturbance (aHR = 1.73, 95% CI: 1.07–2.79, *p* = 0.026) had significantly higher risk of all-cause mortality compared to those who had normal cognitive function and no sleep disturbance (Table 2).

**Table 2:** Associations between cognitive dysfunction and sleep and outcomes. (*n* = 2,690)

Cognitive function and sleep status	All-cause mortality		CVD mortality	
	aHR (95% CI) ^a^	p-value	aHR (95% CI) ^b^	p-value
Model 1 ^a,b^				
C(-), S(-)	ref.		ref.	
C(-), S(+)	1.07 (0.84–1.38)	0.572	0.98 (0.64–1.50)	0.927
C(+), S(-)	**1.59 (1.12–2.26)**	**0.012**	1.53 (0.82–2.88)	0.132
C(+), S(+)	**1.73 (1.07–2.79)**	**0.026**	1.81 (0.98–3.34)	0.057
Model 2 ^c^				
C(-),S(-)	ref.		ref.	
C(-),S(+)	1.06 (0.82–1.38)	0.653	1.00 (0.65–1.54)	0.983
C(+),S(-)	**1.55 (1.08–2.22)**	**0.020**	1.45 (0.75–2.81)	0.265
C(+),S(+)	**1.77 (1.13–2.56)**	**0.014**	1.85 (0.94–3.62)	0.072

Abbreviations: aHR, adjusted hazard ratio; CI, confidence interval; CVD, cardiovascular disease; ref, reference

P-values < 0.05 are shown in bold

^a^ Adjusted for all variables showed significant in the univariate Cox PH regression (except for the laboratory values and variables with excessive missing data), including age (continuous), race, education, BMI, poverty income ratio, cigarette smoking, DM, hypertension, history of CVD, cancer history, CKD, statins, and diuretics

^b^ Adjusted for all variables showed significant in the univariate Cox PH regression (except for the laboratory values and variables with excessive missing data), including age (continuous), race, education, poverty income ratio, DM, hypertension, history of CVD, CKD, statins, and diuretics

^c^ Adjusted for all variables (except for the laboratory values, variables with excessive missing data, and variables that were highly correlated with included factors)

**Fig. 2:**
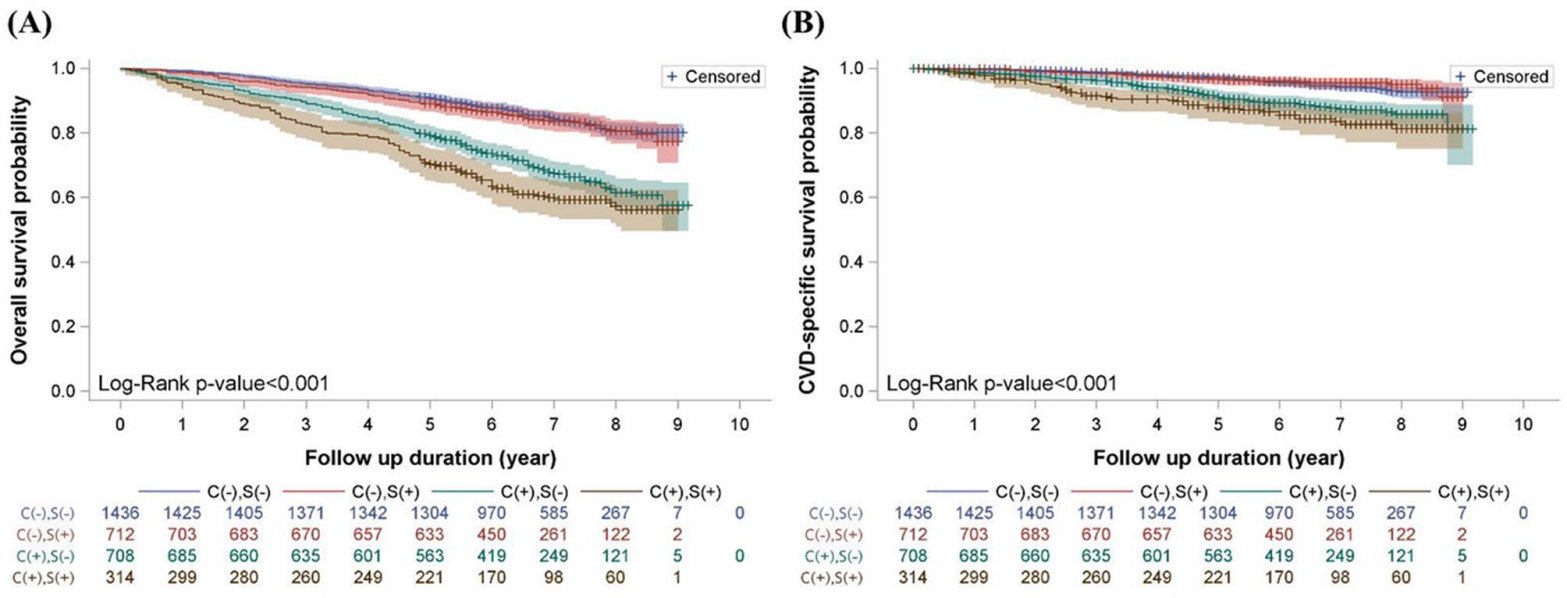
Kaplan–Meier Survival Curves for (**A**) overall survival and (**B**) CVD-specific survival

The Kaplan–Meier survival curves showed a significant difference in overall survival (log-rank p-value < 0.001, Fig. 2A) and CVD-specific survival (log-rank p-value < 0.001, Fig. 2B) among the four groups categorized by cognitive function and sleep status.

### Joint associations between low cognitive function, sleep disturbance, and mortality outcomes, stratified by sex, overweight, obesity, and CKD status

We conducted further subgroup analysis based on sex, BMI status, and CKD status (Table 3).

**Table 3:** Joint associations between cognitive dysfunction, sleep disturbance, and survival outcomes, stratified by sex, obesity status, and CKD status

Subgroup	All-cause mortality		CVD mortality	
	aHR (95% CI) ^a^	p-value	aHR (95% CI) ^b^	p-value
C(-), S(-)	ref.		ref.	
C(-), S(+)	**1.39 (0.99–1.96)**	**0.057**	1.31 (0.62–2.73)	0.466
C(+), S(-)	**1.67 (1.02–2.74)**	**0.044**	1.45 (0.66–3.20)	0.341
C(+), S(+)	**2.50 (1.38–4.56)**	**0.004**	**2.82 (1.20–6.64)**	**0.019**
C(-), S(-)	ref.		ref.	
C(-), S(+)	0.86 (0.56–1.31)	0.457	0.80 (0.43–1.48)	0.464
C(+), S(-)	1.31 (0.88–1.96)	0.179	1.42 (0.62–3.26)	0.399
C(+), S(+)	1.35 (0.72–2.52)	0.343	1.24 (0.57–2.71)	0.585
C(-), S(-)	ref.		ref.	
C(-), S(+)	0.77 (0.41–1.44)	0.399	0.41 (0.09–1.85)	0.234
C(+), S(-)	1.39 (0.72–2.69)	0.322	1.34 (0.66–2.73)	0.403
C(+), S(+)	1.61 (0.81–3.18)	0.165	1.09 (0.36–3.31)	0.876
C(-), S(-)	ref.		ref.	
C(-), S(+)	1.18 (0.86–1.63)	0.298	1.20 (0.67–2.14)	0.533
C(+), S(-)	**1.81 (1.20–2.75)**	**0.007**	1.64 (0.74–3.64)	0.213
C(+), S(+)	**1.93 (1.12–3.31)**	**0.019**	2.28 (0.97–5.36)	0.059
C(-), S(-)	ref.		ref.	
C(-), S(+)	0.98 (0.69–1.38)	0.901	0.75 (0.37–1.53)	0.411
C(+), S(-)	1.47 (0.98–2.20)	0.060	1.58 (0.79–3.16)	0.190
C(+), S(+)	1.53 (0.87–2.70)	0.135	1.10 (0.52–2.35)	0.791
C(-), S(-)	ref.		ref.	
C(-), S(+)	1.07 (0.69–1.66)	0.761	1.24 (0.60–2.56)	0.547
C(+), S(-)	**1.74 (1.04–2.91)**	**0.035**	1.35 (0.55–3.28)	0.501
C(+), S(+)	1.86 (0.95–3.64)	0.070	2.28 (0.97–5.37)	0.060

Abbreviations: aHR, adjusted hazard ratio; BMI, body mass index; CI, confidence interval; CKD, chronic kidney disease; CVD, cardiovascular disease; DM, diabetes mellitus; ref, reference

P-values < 0.05 are shown in bold

^a^ Adjusted for all variables showed significant in the univariate Cox PH regression (except for the laboratory values, variables with excessive missing data, and stratified variable), including age (continuous), race, education, BMI, poverty income ratio, cigarette smoking, DM, hypertension, CVD, cancer history, CKD, statins, and diuretics

^b^ Adjusted for all variables showed significant in the univariate Cox PH regression (except for the laboratory values, variables with excessive missing data, and stratified variable), including age (continuous), race, education, poverty income ratio, DM, hypertension, CVD, CKD, statins, and diuretics

### Sex

Among males, low cognitive function alone (aHR = 1.67, 95% CI: 1.02–2.74, *p* = 0.044) significantly increased the risk of all-cause mortality. The presence of both low cognitive function and sleep disturbances in males significantly increased the risk of both all-cause mortality (aHR = 2.50, 95% CI: 1.38–4.56, *p* = 0.004) and CVD mortality (aHR = 2.82, 95% CI: 1.20–6.64, *p* = 0.019).

### Overweight and obesity status

In the overweight/obese subgroup, low cognitive function significantly increased the risk of all-cause mortality (aHR = 1.81, 95% CI: 1.20–2.75, *p* = 0.007). The presence of both low cognitive function and sleep disturbance was associated with a significantly elevated risk of all-cause mortality (aHR = 1.93, 95% CI: 1.12–3.31, *p* = 0.019).

### CKD status

Among participants diagnosed with established CKD, cognitive dysfunction alone significantly elevated the likelihood of all-cause mortality (adjusted HR = 1.72, 95% CI: 1.03–2.89, *p* = 0.040) (Table 3).

## Discussion

This study examined the combined impact of cognitive dysfunction and sleep disturbances on all-cause and cardiovascular mortality in older adults using NHANES data. Both conditions independently contributed to increased mortality risk, and their coexistence further amplified this association. Individuals with cognitive dysfunction alone had a higher risk of mortality, while those experiencing both cognitive dysfunction and sleep disturbances faced an even greater risk, particularly for cardiovascular-related deaths. As expected, established factors such as age, sex, body mass index, socioeconomic status, and comorbidities—including diabetes mellitus, hypertension, cardiovascular disease, and chronic kidney disease—were also associated with mortality risk. Further stratified analyses suggested that the association between cognitive dysfunction, sleep disturbances, and mortality may differ across subgroups. Among men, the combination of cognitive dysfunction and sleep disturbances was particularly detrimental. In individuals with higher body mass index, cognitive dysfunction alone increased the risk of all-cause mortality, whereas those with both conditions experienced an even higher risk of cardiovascular mortality. Similarly, in individuals with chronic kidney disease, cognitive dysfunction was associated with increased mortality, and the presence of sleep disturbances further exacerbated this risk. These findings are consistent with recent NHANES-based studies in older adults. For example, Zhang et al. [17] demonstrated that diurnal sleep patterns measured by accelerometers were significantly associated with all-cause mortality, while Sakal et al. [18] reported that variability in sleep efficiency was linked to cognitive performance. These studies support the significance of sleep–cognition interactions and further validate the relevance of our findings in geriatric populations. Recognizing cognitive dysfunction and sleep disturbances as independent contributors to mortality risk highlights the need for targeted interventions and early screening strategies to address their compounded effects in older adults. To further assess the robustness of our results, we conducted sensitivity analyses including physical exercise, despite its high proportion of missing data. The associations remained directionally consistent (Supplementary Table 3), reinforcing the validity of our conclusions.

This study is among the first to examine the combined effects of cognitive impairment and sleep disturbances on all-cause and cardiovascular mortality, highlighting their synergistic impact. Prior studies have tended to investigate the impact of either abnormal sleep patterns or cognitive function on all-cause or CVD-related death [15, 19–22]. Similar to our results, these studies found that disruption in sleep and/or abnormal sleep patterns were associated with increased mortality [15, 19, 20]. Other studies have found cognitive dysfunction, including mild cognitive impairment, increases mortality risk [21–23]. One study found that the risk of mortality in patients with mild cognitive impairment was higher in males, and in participants with higher education (> 12 years), history of heart disease, and who did not exercise [22]. Another study found that rapid cognitive decline was associated with a 75% higher risk of death, especially in participants aged 65 to 79 years and those with normal cognitive function at baseline [23].

The combined association of abnormal sleep patterns and cognitive dysfunction with mortality suggests that these conditions may jointly contribute to increased risk. Several prior studies have identified potential links between sleep disturbances and cognitive dysfunction. Specifically, abnormal sleep patterns, including insomnia, fragmentation, sleep behavior disorders, and excessive time in bed, have been associated with decline in cognitive function [11, 13, 14, 24]. It is not entirely clear how abnormal sleep causes loss of cognitive function or vice versa. Several studies have indicated a bidirectional relationship between sleep and dementia, particularly in Alzheimer’s disease (AD) [11, 13]. Sleep disorders, including disruption of neuronal rhythm activity and circadian disturbances, are considered as both candidate risks and consequences of AD [13]. Sleep disruption appears to impact complex neuronal circuitry resulting in disruption of different cognitive functions such as memory consolidation, attention performance and alertness [11]. Understanding this interaction is of great importance to public health as insomnia and cognitive decline affect large segments of the population [2, 3, 7].

The synergism between disturbed sleep patterns and cognition on mortality may stem from shared biological mechanisms, such as chronic inflammation, hormonal dysregulation, impaired neurovascular function, abnormal amyloid (Aβ) deposition, and exacerbated oxidative stress [11, 25, 26]. Oxidative stress damages cells and tissues including vascular walls and neurons, contributing to both cardiovascular and neurodegenerative processes [6]. Sleep disturbances could accelerate cognitive decline, and cognitive dysfunction may further disrupt sleep-wake cycles, creating a feedback loop that worsens health outcomes [25]. Both cognitive impairment and sleep disturbances activate the body’s stress response systems, including the hypothalamic-pituitary-adrenal (HPA) axis [25]. Chronic activation of this system can lead to elevated levels of stress hormones like cortisol, which are linked to cardiovascular disease risk and poor health outcomes. Poor sleep quality and cognitive impairment can also disrupt autonomic nervous system balance, leading to increased sympathetic activity and reduced parasympathetic activity [27]. This imbalance can contribute to hypertension, increased heart rate, and other cardiovascular risk factors [27]. In addition, sleep disturbances and cognitive dysfunction are independently associated with increased inflammatory markers such as c-reactive protein (CRP) and interleukin-6 (IL-6) [28]. Systemic inflammation is viewed as an early event in the course of Alzheimer’s disease [28]. Sleep disorders may also promote atherosclerosis and endothelial dysfunction, increasing vascular risk [29]. Since both sleep dysfunction and cognitive impairment can compromise cerebral blood flow, they might jointly accelerate brain aging and cognitive decline, further increasing the risk of both neurodegenerative and cardiovascular conditions.

The synergistic effect observed between sleep and cognitive function in our study may support the concept of a positive correlation between sleep quality and cognitive status. Recently, a new theory has emerged suggesting that good sleep—particularly REM sleep—enhances glymphatic clearance in the brain [30, 31]. The glymphatic system is a “pseudo-lymphatic” perivascular network in the brain that is responsible for removing harmful interstitial metabolic waste products. Clearance of the glymphatic system can be altered by lack of sleep; sleep promotes the ability of the glymphatic system to remove brain waste solutes, particularly during deep and REM sleep stages [30, 31]. A large literature review found that glymphatic clearance plays a major role in Alzheimer’s disease [30]. It also concluded that it is during sleep that the majority of glymphatic waste clearance occurs, and lifestyle choices such as sleep position, alcohol intake, exercise, 3-omega consumption, intermittent fasting, and chronic stress all influence glymphatic clearance [30].

In summary, this study highlights the critical need for routine assessment and management of sleep disorders in older adults with cognitive impairment to identify those at increased risk of mortality. Healthcare providers should prioritize comprehensive screening and targeted interventions that focus on improving sleep quality in this vulnerable population. Evidence-based strategies, such as cognitive-behavioral therapy for insomnia (CBT-I) and tailored sleep hygiene education, may play a role in mitigating the adverse effects of sleep disturbances on cognitive health and overall well-being. Further research is warranted to develop and validate integrative approaches that address sleep disorders in cognitively impaired individuals, ultimately improving health outcomes and quality of life.

### Strengths and limitations

This study benefits from a large and nationally representative sample provided by NHANES, lending significant generalizability to the findings across the broader U.S. adult population. The utilization of standardized cognitive assessments and detailed data on demographic, health, and sleep-related variables enabled a thorough analysis of the relationships between cognitive dysfunction, sleep disturbances, and mortality outcomes. Furthermore, the stratified analysis based on factors such as sex, BMI, and chronic kidney disease offers valuable insights into how these variables modulate mortality risk. Several limitations should be acknowledged in interpreting our findings. First, the reliance on self-reported data for sleep disturbances may introduce recall or reporting bias, potentially leading to misclassification and introducing potential selection bias in the study. Due to the process of the missing values, some important variable may be excluded, such as physical activity. Objective sleep assessments, such as polysomnography or actigraphy, would provide more accurate evaluations of sleep disturbances and their impact on cognitive function and mortality risk. Nevertheless, the retrospective design and inherent limitations of the database precluded access to detailed measurement data. Variables such as employment status and the use of sleep-inducing medications were not included in the survey. Additionally, cognitive function was assessed using a limited set of standardized tests, which, while widely used, may not capture the full spectrum of cognitive impairment, particularly in domains such as executive function or processing speed. Expanding cognitive assessments to include a broader range of neuropsychological measures could enhance the comprehensiveness of future studies. Furthermore, the absence of data on REM and NREM sleep stages limits our ability to differentiate how distinct aspects of sleep architecture may differentially contribute to cognitive dysfunction and mortality risk. Future research incorporating detailed sleep metrics would allow for a more nuanced understanding of these relationships. Future studies should integrate depression assessment to explore its potential modifying role and better elucidate the interplay between these conditions and mortality risk. Addressing these limitations through more comprehensive investigations will help strengthen causal inferences and expand the clinical relevance of these findings.

## Conclusions

In conclusion, this study highlights the significant impact of cognitive dysfunction and sleep disturbances on both all-cause and cardiovascular mortality among older adults. The findings indicate that individuals with either condition alone face increased mortality risks, and having both conditions further amplifies this risk. Given the aging global population and the prevalence of cognitive impairments and sleep issues, our results emphasize the urgent need for comprehensive strategies that address these conditions. Implementing interventions that enhance cognitive health and improve sleep quality could potentially reduce mortality rates and improve quality of life for this vulnerable population. Future research should continue to explore the mechanisms underlying these associations to better inform clinical and public health interventions.

## Supplementary Information

Below is the link to the electronic supplementary material.

Supplementary Material 1

## Data Availability

The datasets used and/or analysed during the current study are available from the corresponding author on reasonable request.

## Abbreviations

CERAD: Consortium to Establish a Registry for Alzheimer’s Disease
aHR: Adjusted hazard ratios
NHANES: National Health and Nutrition Examination Survey
MCI: Mild cognitive impairment
MEC: Mobile examination center
