# Supplementary material for: Combined effect of cognitive dysfunction and sleep disturbance on mortality risk: NHANES 2011–2014
Source: Ann Gen Psychiatry. 2025 Sep 2;24:52. doi: 10.1186/s12991-025-00593-7 (PMC12406422; doi:10.1186/s12991-025-00593-7)
Supplement: Supplementary file 1 — Supplementary Material 1 [file 12991_2025_593_MOESM1_ESM.docx]

**Supplemental Table 1.** Associations between cognitive dysfunction, sleep disturbance, and all-cause mortality, univariate Cox PH regression.

|  | **All-cause mortality** | | | | | |  |
| --- | --- | --- | --- | --- | --- | --- | --- |
|  | HR (95% CI) | | p-value | | Overall p-value | |  |
| **Cognitive function and sleep status** |  | |  | | **<0.001** | |  |
| C(-), S(-) | ref. | |  | |  | |  |
| C(-), S(+) | 1.10 (0.83-1.46) | | 0.483 | |  | |  |
| C(+), S(-) | **3.15 (2.32-4.26)** | | **<0.001** | |  | |  |
| C(+), S(+) | **3.90 (2.71-5.60)** | | **<0.001** | |  | |  |
| **Age, years** | **1.14 (1.13-1.16)** | | **<0.001** | | **<0.001** | |  |
| **Sex** |  | |  | | 0.105 | |  |
| Male | ref. | |  | |  | |  |
| Female | 0.82 (0.64-1.05) | | 0.105 | |  | |  |
| **Race/ethnicity** |  | |  | | **0.024** | |  |
| Non-Hispanic White | ref. | |  | |  | |  |
| Non-Hispanic Black | 1.06 (0.83-1.36) | | 0.638 | |  | |  |
| Hispanic | **0.70 (0.49-0.99)** | | **0.046** | |  | |  |
| Others | 0.72 (0.50-1.04) | | 0.080 | |  | |  |
| **Education** |  | |  | | **0.008** | |  |
| High school and above | ref. | |  | |  | |  |
| Never attend high school | **1.54 (1.13-2.10)** | | **0.008** | |  | |  |
| **BMI, kg/m^2^** |  | |  | | **0.044** | |  |
| Normal | ref. | |  | |  | |  |
| Underweight | **2.34 (1.17-4.68)** | | **0.018** | |  | |  |
| Overweight | 0.88 (0.65-1.20) | | 0.411 | |  | |  |
| Obese | 0.91 (0.70-1.18) | | 0.463 | |  | |  |
| **Poverty income ratio** |  | |  | | **0.031** | |  |
| Not poor | ref. | |  | |  | |  |
| Poor | **1.46 (1.04-2.05)** | | **0.031** | |  | |  |
| **Cigarette smoking** |  | |  | | **0.015** | |  |
| Never | ref. | |  | |  | |  |
| Former | **1.44 (1.14-1.83)** | | **0.004** | |  | |  |
| Current smoker | 1.29 (0.83-2.00) | | 0.256 | |  | |  |
| **Depression** | |  | |  | | 0.439 | |
| No | | ref. | |  | |  | |
| Yes | | 1.18 (0.77-1.80) | | 0.439 | |  | |
| **DM** |  | |  | | **<0.001** | |  |
| No | ref. | |  | |  | |  |
| Yes | **1.61 (1.27-2.03)** | | **<0.001** | |  | |  |
| **Hypertension** |  | |  | | **<0.001** | |  |
| No | ref. | |  | |  | |  |
| Yes | **1.88 (1.55-2.28)** | | **<0.001** | |  | |  |
| **CVD** |  | |  | | **<0.001** | |  |
| No | ref. | |  | |  | |  |
| Yes | **2.47 (2.01-3.03)** | | **<0.001** | |  | |  |
| **Cancer history** |  | |  | | **<0.001** | |  |
| No | ref. | |  | |  | |  |
| Yes | **1.51 (1.23-1.86)** | | **<0.001** | |  | |  |
| **CKD** |  | |  | | **<0.001** | |  |
| No | ref. | |  | |  | |  |
| Yes | **2.66 (2.18-3.26)** | | **<0.001** | |  | |  |
| **Statins** |  | |  | | **<0.001** | |  |
| No | ref. | |  | |  | |  |
| Yes | **1.31 (1.14, 1.51)** | | **<0.001** | |  | |  |
| **Diuretics** |  | |  | | **<0.001** | |  |
| No | ref. | |  | |  | |  |
| Yes | **2.17 (1.81-2.59)** | | **<0.001** | |  | |  |

Abbreviations: BMI, body mass index; CKD, chronic kidney disease; CI, confidence interval; CVD, cardiovascular disease; DM, diabetes mellitus; HR, hazard ratio; ref, reference

P-values <0.05 are shown in bold.

**Supplemental Table 2.** Associations between cognitive dysfunction and sleep and CVD-related mortality univariate Cox PH regression.

|  | **CVD-related mortality** | | | | | | | |
| --- | --- | --- | --- | --- | --- | --- | --- | --- |
|  | HR (95% CI) | | p-value | | | | Overall p-value | |
| **Cognitive dysfunction and sleep** |  | |  | | | | **<0.001** | |
| C(-), S(-) | ref. | |  | | | |  | |
| C(-), S(+) | 1.08 (0.74-1.59) | | 0.676 | | | |  | |
| C(+), S(-) | **3.71 (2.43-5.66)** | | **<0.001** | | | |  | |
| C(+), S(+) | **4.63 (2.86-7.50)** | | **<0.001** | | | |  | |
| **Age, years** | **1.18 (1.14-1.22)** | | **<0.001** | | | | **<0.001** | |
| **Sex** |  | |  | | | | 0.157 | |
| Male | ref. | |  | | | |  | |
| Female | 0.78 (0.54-1.11) | | 0.157 | | | |  | |
| **Race/ethnicity** |  | |  | | | | **0.014** | |
| Non-Hispanic White | ref. | |  | | | |  | |
| Non-Hispanic Black | 1.30 (0.86-1.96) | | 0.211 | | | |  | |
| Hispanic | **0.59 (0.37-0.93)** | | **0.024** | | | |  | |
| Others | 0.76 (0.42-1.37) | | 0.346 | | | |  | |
| **Education** |  | |  | | | | **0.010** | |
| High school and above | ref. | |  | | | |  | |
| Never attend high school | **2.00 (1.20-3.32)** | | **0.010** | | | |  | |
| **BMI, kg/m^2^** |  | |  | | | | 0.458 | |
| Normal | ref. | |  | | | |  | |
| Underweight | 2.60 (0.63-10.76) | | 0.180 | | | |  | |
| Overweight | 0.90 (0.59-1.37) | | 0.613 | | | |  | |
| Obese | 0.94 (0.58-1.53) | | 0.806 | | | |  | |
| **Poverty income ratio** |  | |  | | | | **0.017** | |
| Not poor | ref. | |  | | | |  | |
| Poor | **1.84 (1.12-3.01)** | | **0.017** | | | |  | |
| **Cigarette smoking** |  | |  | | | | 0.553 | |
| Never | ref. | |  | | | |  | |
| Former | 1.18 (0.81-1.72) | | 0.381 | | | |  | |
| Current smoker | 0.92 (0.44-1.90) | | 0.807 | | | |  | |
| **Depression** | |  | |  | | 0.291 | | |
| No | | ref. | |  | |  | | |
| Yes | | 1.33 (0.77-2.31) | | 0.291 | |  | | |
| **DM** |  | |  | | | | **<0.001** | |
| No | ref. | |  | | | |  | |
| Yes | **1.97 (1.44-2.70)** | | **<0.001** | | | |  | |
| **Hypertension** |  | |  | | | | **<0.001** | |
| No | ref. | |  | | | |  | |
| Yes | **2.42 (1.64-3.57)** | | **<0.001** | | | |  | |
| **CVD** |  | |  | | | | **<0.001** | |
| No | ref. | |  | | | |  | |
| Yes | **4.15 (3.27-5.28)** | | **<0.001** | | | |  | |
| **Cancer history** |  | |  | | | | 0.420 | |
| No | ref. | |  | | | |  | |
| Yes | 1.17 (0.79-1.74) | | 0.420 | | | |  | |
| **CKD** |  | |  | | | | **<0.001** | |
| No | ref. | |  | | | |  | |
| Yes | **3.95 (2.76-5.65)** | | **<0.001** | | | |  | |
| **Statins** |  | |  | | **0.008** | | |  |
| No | ref. | |  | |  | | |  |
| Yes | **1.48 (1.12-1.95)** | | **0.008** | |  | | |  |
| **Diuretics** |  | |  | | **<0.001** | | |  |
| No | ref. | |  | |  | | |  |
| Yes | **2.99 (2.15-4.16)** | | **<0.001** | |  | | |  |

Abbreviations: CVD, cardiovascular disease; DM, diabetes mellitus; HR, hazard ratio; CI, confidence interval; ref, reference; BMI, body mass index; CKD, chronic kidney disease.

P-values < 0.05 are shown in bold.

**Supplemental Table 3.** Associations between cognitive dysfunction and sleep and outcomes. (include physical exercise)

| Cognitive function and sleep status | All-cause mortality | |  | CVD mortality | |
| --- | --- | --- | --- | --- | --- |
|  | aHR (95% CI) ^a^ | p-value |  | aHR (95% CI) ^b^ | p-value |
| Model 1C |  |  |  |  |  |
| C(-), S(-) | ref. |  |  | ref. |  |
| C(-), S(+) | 1.07 (0.82-1.39) | 0.606 |  | 1.00 (0.64-1.56) | 0.990 |
| C(+), S(-) | **1.55 (1.09-2.21)** | **0.016** |  | 1.50 (0.80-2.84) | 0.199 |
| C(+), S(+) | **1.70 (1.06-2.73)** | **0.030** |  | 1.82 (0.99-3.37) | 0.055 |
| Model 1D |  |  |  |  |  |
| C(-),S(-) | ref. |  |  | ref. |  |
| C(-),S(+) | 1.02 (0.69-1.51) | 0.919 |  | 0.65 (0.31-1.39) | 0.258 |
| C(+),S(-) | 1.20 (0.70-2.06) | 0.496 |  | 0.95 (0.37-2.43) | 0.907 |
| C(+),S(+) | 1.10 (0.44-2.75) | 0.838 |  | 0.79 (0.20-3.16) | 0.736 |

Abbreviations: aHR, adjusted hazard ratio; CI, confidence interval; CVD, cardiovascular disease; ref, reference

P-values <0.05 are shown in bold.

Model 1C including missing value of physical exercise. Model 1D excluding missing value of physical exercise.

^a^ Adjusted for all variables showed significant in the univariate Cox PH regression (except for the laboratory values and variables with excessive missing data), including age (continuous), race, education, physical exercise, BMI, poverty income ratio, cigarette smoking, DM, hypertension, history of CVD, cancer history, CKD, statins, and diuretics.

^b^ Adjusted for all variables showed significant in the univariate Cox PH regression (except for the laboratory values and variables with excessive missing data), including age (continuous), race, education, physical exercise, poverty income ratio, DM, hypertension, history of CVD, CKD, statins, and diuretics.
